# Mixedness, Coherence and Entanglement in a Family of Three-Qubit States

**DOI:** 10.3390/e24030324

**Published:** 2022-02-24

**Authors:** Joanna K. Kalaga, Wiesław Leoński, Radosław Szczȩśniak, Jan Peřina

**Affiliations:** 1 Quantum Optics and Engineering Division, Institute of Physics, University of Zielona Góra, Prof. Z. Szafrana 4a, 65-516 Zielona Góra, Poland; j.kalaga@if.uz.zgora.pl; 2 Division of Physics, Czȩstochowa University of Technology, Ave. Armii Krajowej 19, 42-200 Czȩstochowa, Poland; radoslaw.szczesniak@pcz.pl; 3 Joint Laboratory of Optics of Palacký University and Institute of Physics of CAS, Faculty of Science, Palacký University, 17. listopadu 12, 771 46 Olomouc, Czech Republic; jan.perina.jr@upol.cz

**Keywords:** quantum entanglement, linear entropy, coherence, purity of states, concurrence, three-qubit systems

## Abstract

We consider a family of states describing three-qubit systems. We derived formulas showing the relations between linear entropy and measures of coherence such as degree of coherence, first- and second-order correlation functions. We show that qubit–qubit states are strongly entangled when linear entropy reaches some range of values. For such states, we derived the conditions determining boundary values of linear entropy parametrized by measures of coherence.

## 1. Introduction

Recent developments in modern physics showed that quantum correlations such as quantum entanglement and their relations to quantum coherence play a valid role in understanding the nature of various physical systems.

Coherence is a phenomenon studied not only in classical theories such as ray optics but also is discussed for a variety of quantum systems, for instance, those related to quantum information theory. For the first time, the concept of the degree of coherence was introduced in the area of classical field propagation theory by Zernike in 1938 [1]. Next, in 1950, Hanbury Brown and Twiss investigated the higher-order coherence in the stellar interferometer system [2]. The quantum coherence theory was formulated in 1963 by Glauber [3,4] and Sudarshan [5] and then developed in 1965 by Metha and Sudarshan [6]. On the other hand, we can find an exhaustive presentation of classical and quantum coherence theory in [7] and [8,9], respectively. The quantum coherence theory found numerous applications in research in the field of quantum optics [3,4]. Primarily, in recent years, the relations between quantum coherence and entanglement have been investigated in various models, including those describing atomic ensembles in high-Q cavities [10], optomechanical systems [11], two strongly coupled bosonic modes [12], or three-mode optomechanical systems [13].

The entangled systems found various implementations in the quantum information theory, especially in quantum communication, quantum cryptography [14], and quantum computations [15,16,17,18,19,20,21,22]. The maximally or strongly entangled states play a fundamental role in such processes as quantum teleportation [23,24,25,26] or secure quantum communication [27,28]. Thus, it is still essential to deepen knowledge about the nature of entanglement and its relations to other forms of quantum correlations and coherence. Thus, in our research, we will not only consider the relations between entanglement and coherence but also the mixedness of states. The mutual relations between the quantities describing entanglement and mixedness [29,30,31,32,33,34,35] or coherence and mixedness [36,37,38,39,40,41], or coherence and entanglement [42,43,44,45,46,47,48] have already been studied in recent years. Our research concerns a three-qubit model that can be implemented in various physical systems. For instance, it could be three two-state spin mutually interacting systems [49] or three two-level atoms [50,51]. In fact, all tripartite systems for which evolution remains closed within a finite set of the states (here, to two states) could be considered in that context. Therefore, our studies are more general, and obtained results can be used in various physical systems.

The paper is organized as follows: in Section 2, we introduce two families of states describing the three-qubit systems of our interest. For such defined groups of states, in Section 3, we study the relations between the mixedness defined by linear entropy and coherence for a qubit–qubit subsystem of our tripartite model. Applying entanglement measures, we find the conditions determining when strongly entangled mixed states appear for the qubit–qubit subsystems. In Section 4 and Section 5, for the double excited systems, we analyze the first- and second-order correlation functions, respectively. For two-qubit states, we find possible values of linear entropy parametrized by both correlation functions considered here and derive the formulas which allow identifying ranges of values of discussed parameters for which strongly entangled states can be found.

## 2. The Three-Qubit System

**Figure 1:**
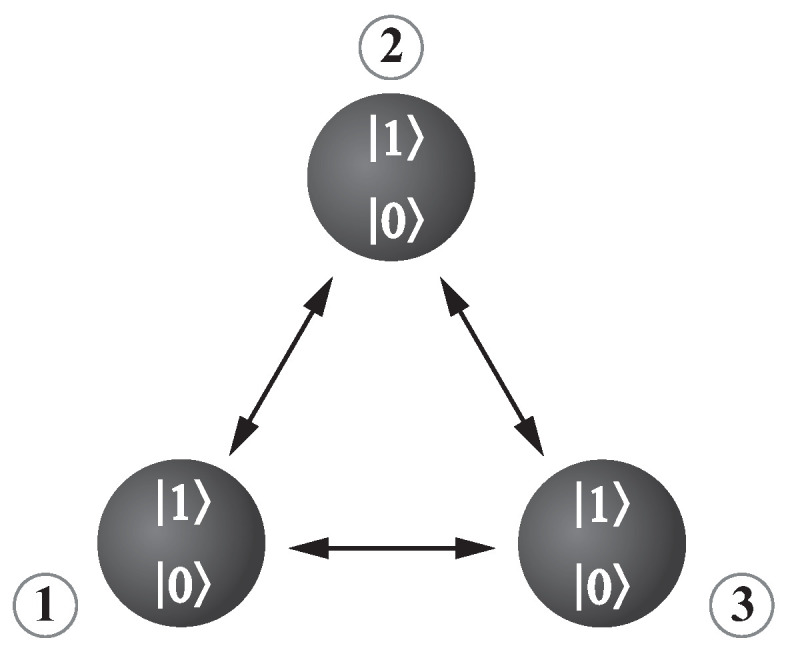
The model of a three-qubit system. The qubits are represented by the black circles and the arrows symbolize the analyzed here the bipartite correlations.

In this paper, we concentrate on the states describing three-qubit systems (see Figure 1) and studying relations among various quantities describing two-qubit correlations and mixedness of states. The presented analysis is devoted to the bosonic systems that can behave as linear or nonlinear quantum scissors [52]. In other words, the wave function describing the states of such systems is defined in the finite-dimensional Hilbert space [53,54]. Here, we discuss a particular case when only two states are populated for each subsystem. For instance, in the cases of quantum-optical systems, they are vacuum |0〉 and one-photon |1〉 states. However, we do not analyze a specific quantum model, but we examine the various states generated in such systems.

In particular, we shall focus on the two families of states: those corresponding to one excitation in the system and, next, two excitations. First, we concentrate on the situation when we deal with a single excitation, so the total number of photons/phonons $\langle n\rangle =\langle n_{1}\rangle +\langle n_{2}\rangle +\langle n_{3}\rangle =1$, where indices 1–3 label the qubits. For such a case, the wave function describing the system’s state is

(1) $$|\psi \rangle =C_{001}|001\rangle +C_{010}|010\rangle +C_{100}|100\rangle \;,$$

and the corresponding density matrix takes the following form:

(2) $$\rho =\left|\psi \rangle \langle \psi \right|=\left[\begin{array}{cccccccc} 0 & 0 & 0 & 0 & 0 & 0 & 0 & 0\\ 0 & P_{001} & C_{001}^{*}C_{010} & 0 & C_{001}^{*}C_{100} & 0 & 0 & 0\\ 0 & C_{010}^{*}C_{001} & P_{010} & 0 & C_{010}^{*}C_{100} & 0 & 0 & 0\\ 0 & 0 & 0 & 0 & 0 & 0 & 0 & 0\\ 0 & C_{100}^{*}C_{001} & C_{100}^{*}C_{010} & 0 & P_{100} & 0 & 0 & 0\\ 0 & 0 & 0 & 0 & 0 & 0 & 0 & 0\\ 0 & 0 & 0 & 0 & 0 & 0 & 0 & 0\\ 0 & 0 & 0 & 0 & 0 & 0 & 0 & 0 \end{array}\right].$$

The $C_{ijk}$ are the complex probability amplitudes corresponding to the states |ijk〉, whereas $P_{ijk}=C_{ijk}^{*}C_{ijk}$ are the probabilities related to the latter.

For the second situation that we are interested in, two excitations are present in the system – $\langle n\rangle =\langle n_{1}\rangle +\langle n_{2}\rangle +\langle n_{3}\rangle =2$. For such a case, we consider the following wave-function:

(3) $$|\psi \rangle =C_{011}|011\rangle +C_{101}|101\rangle +C_{110}|110\rangle \;,$$

and the corresponding density matrix

(4) $$\rho =\left|\psi \rangle \langle \psi \right|=\left[\begin{array}{cccccccc} 0 & 0 & 0 & 0 & 0 & 0 & 0 & 0\\ 0 & 0 & 0 & 0 & 0 & 0 & 0 & 0\\ 0 & 0 & 0 & 0 & 0 & 0 & 0 & 0\\ 0 & 0 & 0 & P_{011} & 0 & C_{011}^{*}C_{101} & C_{011}^{*}C_{110} & 0\\ 0 & 0 & 0 & 0 & 0 & 0 & 0 & 0\\ 0 & 0 & 0 & C_{101}^{*}C_{011} & 0 & P_{101} & C_{101}^{*}C_{110} & 0\\ 0 & 0 & 0 & C_{110}^{*}C_{011} & 0 & C_{110}^{*}C_{101} & P_{110} & 0\\ 0 & 0 & 0 & 0 & 0 & 0 & 0 & 0 \end{array}\right].$$

The two families of states analyzed here are three-qubit states and belong to the same class—that of *W*-states (for the discussion of various classes of three-qubit states, see [55,56,57] *and the references quoted therein*). Despite this fact, as we shall show, the values of the first and second-order correlation functions allow for discriminating the states from the two families. Thus, those parameters behave differently from the concurrence and degree of coherence, where those two parameters do not allow for such discrimination. From the other side, the states considered here are those involving one or two excitations. Such states could be physically generated by the systems called *quantum scissors* (both linear and nonlinear ones) [52], and, thus, they seem to be interesting from the practical point of view.

Due to the great attention recently given to *W*-states [58,59,60,61,62,63,64] and a broad range of their application in quantum information systems, we shall focus here on two types of such states. *W*-states can be employed, for instance, in quantum teleportation systems [65,66,67], dense coding [68,69,70], and cryptographic protocols [71,72].

## 3. The Linear Entropy and Degree of Coherence

In our studies, we concentrate on finding the relation among various quantities characterizing bipartite systems, being subsystems of our three-qubit model. Such two-qubit subsystems appear to be in mixed states. Therefore, one of the quantities analyzed by us is the degree of mixedness. As a measure of mixedness, we will apply the linear entropy defined with the application of purity parameter [31]

(5) $$E\left(\rho \right)\equiv \frac{Dim}{Dim-1}\left[1-Tr\left(\rho^{2}\right)\right]\;,$$

where Dim denotes the dimension of ρ. In our studies, we analyze the mixedness of two-qubit states. Therefore, we assume that Dim=4 and thus the *linear entropy* can be written as:

(6) $$E_{ij}=E\left(\rho_{ij}\right)\equiv \frac{4}{3}\left[1-Tr\left(\rho_{ij}^{2}\right)\right].$$

where $\rho_{ij}$ is the reduced density matrix describing the two-qubit state.

Next, we will analyze the coherence. In this paper, we will study two manifestations of that phenomenon. Firstly, we concentrate on the internal coherence of any two subsystems (from all three), described by the *degree of coherence*. In the next section, we will focus on the mutual coherence—*cross-coherence*.

The degree of coherence that will be applied here can be defined with an application of the degrees of first-order coherence $D_{i}$ and $D_{j}$ corresponding to the qubits *i* and *j*

(7) $$D_{k}=\sqrt{2\mathrm{Tr}\left(\rho_{k}^{2}\right)-1},\;k=i,j=\left\{1,2,3\right\}\;,$$

where $\rho_{k}$ is the reduced density matrix related to qubit *k*. Next, the parameter $D_{k}$ is used to define the *degree of coherence*$D_{ij}^{2}$ in the bipartite system [9,73]:

(8) $$D_{ij}^{2}=\left(D_{i}^{2}+D_{j}^{2}\right)/2.$$

The quantity $D_{ij}^{2}$ can be treated as a measure of the total coherence inside the two independently considered subsystems. Thus, $D_{ij}^{2}$ is equal to 0 only if both subsystems show no coherence. The states with $D_{ij}^{2}=0$ are the state that gives maximal violation of the CHSH inequality—the Bell states [73].

**Figure 2:**
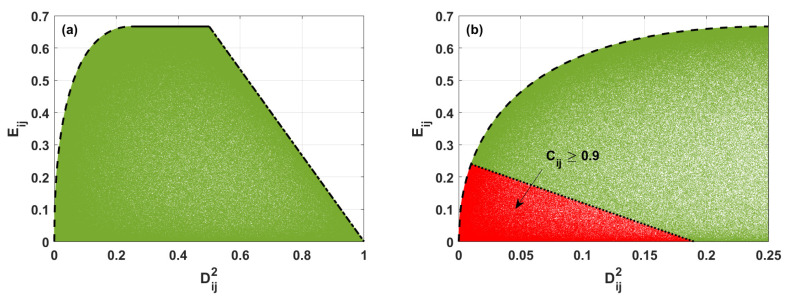
(**a**) Linear entropy $E_{ij}$ versus degree of coherence $D_{ij}^{2}$ for two-qubit states described by the density matrix $\rho_{ij}$, found numerically (green area). Black lines are plotted according to the analytical formulas derived here determining the borders between various regions of the states. (**b**) The same as in (**a**). Additionally, the red area presents the possible values of linear entropy and degree of coherence for two-qubit states with concurrence $C_{ij}>0.9$ (red area).

To find the relations between the values of linear entropy and the degree of coherence for two-qubit mixed states, we have generated $10^{6}$ random three-qubit states defined by the density matrix ρ (2). Next, we have found a reduced density matrix $\rho_{ij}$ representing the two-qubit states discussed by us. Such matrices were derived from the full three-qubit density matrix by tracing out one subsystem—the qubit *k*. Next, for each qubit–qubit state, we have calculated both linear entropy $E(\rho_{ij})$ and degree of coherence $D_{ij}^{2}$. The results showing how the value of linear entropy depends on the values of the degree of coherence for the system involving single excitations are presented in Figure 2. It is interesting that those results are identical to those corresponding to the systems with two excitations and described by the density matrix defined by Equation (4). This is the consequence of the fact that, since the states (2) can be transformed into states (4) by a local unitary transformation, linear entropy and degree of coherence are invariant quantities under a local unitary transformation.

For two-qubit mixed states, we see that, for a given value of $D_{ij}^{2}$, the linear entropy reaches only some values represented in Figure 2 by the green area. Moreover, the black lines appearing in Figure 2 correspond to the boundary values of $E_{ij}$ defined by Equations (17), (20), and (24).

To find the upper bound of the degree of mixedness for two-qubit states, we express $E_{ij}$ and $D_{ij}^{2}$ for each pair of qubits by the probabilities $P_{ijk}$. For the system described by the density matrix ρ (2), the entropy and degree of coherence are given by (for more details of the calculation method, see in [34,74]):

(9) $$\begin{array}{ccc} E_{12} & \equiv & \frac{8}{3}\left(-P_{100}^{2}+P_{100}-P_{010}^{2}+P_{010}-2P_{100}P_{010}\right)\;,\\ E_{13} & \equiv & \frac{8}{3}\left(-P_{100}^{2}+P_{100}-P_{001}^{2}+P_{001}-2P_{100}P_{001}\right)\;,\\ E_{23} & \equiv & \frac{8}{3}\left(-P_{010}^{2}+P_{010}-P_{001}^{2}+P_{001}-2P_{010}P_{001}\right)\;, \end{array}$$

(10) $$\begin{array}{ccc} D_{12}^{2} & = & 1+2\left(P_{100}^{2}-P_{100}+P_{010}^{2}-P_{010}\right)\;,\\ D_{13}^{2} & = & 1+2\left(P_{100}^{2}-P_{100}+P_{001}^{2}-P_{001}\right)\;,\\ D_{23}^{2} & = & 1+2\left(P_{010}^{2}-P_{010}+P_{001}^{2}-P_{001}\right), \end{array}$$

whereas, for the double excited system, the formulas describing $E_{ij}$ and $D_{ij}^{2}$ take the following forms:

(11) $$\begin{array}{ccc} E_{12} & \equiv & \frac{8}{3}\left(-P_{011}^{2}+P_{011}-P_{101}^{2}+P_{101}-2P_{011}P_{101}\right)\;,\\ E_{13} & \equiv & \frac{8}{3}\left(-P_{011}^{2}+P_{011}-P_{110}^{2}+P_{110}-2P_{011}P_{110}\right)\;,\\ E_{23} & \equiv & \frac{8}{3}\left(-P_{110}^{2}+P_{110}-P_{101}^{2}+P_{101}-2P_{110}P_{101}\right)\;, \end{array}$$

(12) $$\begin{array}{ccc} D_{12}^{2} & = & 1+2\left(P_{101}^{2}-P_{101}+P_{011}^{2}-P_{011}\right)\;,\\ D_{13}^{2} & = & 1+2\left(P_{011}^{2}-P_{011}+P_{110}^{2}-P_{110}\right)\;,\\ D_{23}^{2} & = & 1+2\left(P_{101}^{2}-P_{101}+P_{110}^{2}-P_{110}\right)\;. \end{array}$$

When $D_{ij}^{2}\in \langle 0;0.25\rangle$, the maximal values of linear entropy are represented in Figure 2 by the black dashed line. The two-qubit states maximizing the linear entropy for a given value of the degree of coherence are the Werner states. Such states are mixtures of the Bell states and separable ones. The density matrix corresponding to the Werner states discussed here and corresponding to the single excitation’s case can be written as:

(13) $$\rho_{W}=\left[\begin{array}{cccc} 1-\alpha & 0 & 0 & 0\\ 0 & \alpha /2 & \alpha /2 & 0\\ 0 & \alpha /2 & \alpha /2 & 0\\ 0 & 0 & 0 & 0 \end{array}\right]\;,$$

whereas, for systems with two excitations, has the form:

(14) $$\rho_{W}=\left[\begin{array}{cccc} 0 & 0 & 0 & 0\\ 0 & \alpha /2 & \alpha /2 & 0\\ 0 & \alpha /2 & \alpha /2 & 0\\ 0 & 0 & 0 & 1-\alpha \end{array}\right]\;,$$

and the wave-function describing such states is

(15) $$|\psi \rangle =\sqrt{\alpha /2}|\psi_{1}\rangle +\sqrt{\alpha /2}|\psi_{2}\rangle +\sqrt{1-\alpha }|\psi_{3}\rangle ,$$

where $\psi_{i}=\{|001\rangle ,|010\rangle ,|100\rangle \}$ and $\psi_{i}=\{|011\rangle ,|101\rangle ,|110\rangle \}$ for the system with single and double excitation, respectively. The parameter α is related to the probabilities of finding the system in one of these states. Thus, using α, $E_{ij}$ and $D_{ij}^{2}$ can be expressed as:

(16) $$\begin{array}{ccc} E_{ij} & = & \frac{8}{3}\left(\alpha -\alpha^{2}\right)\;,\\ D_{ij}^{2} & = & 2\left(\frac{\alpha^{2}}{2}-\alpha \right)+1\;. \end{array}$$

From Equations (16), we obtain the maximal values of linear entropy for $D_{ij}^{2}\in \langle 0;0.25\rangle$ (the black dashed line in Figure 2)

(17) $$E_{ij}=-\frac{8}{3}\left(D_{ij}^{2}-\sqrt{\left(D_{ij}^{2}\right)}\right).$$

In Figure 2, the solid black line represents the maximal value of $E_{ij}$ when $D_{ij}^{2}\in \langle 0.25;0.5\rangle$. For such a case, the reduced density matrix $\rho_{ij}$ for the system with a single excitation takes the following form:

(18) $$\rho_{ij}=\left[\begin{array}{cccc} 1/2 & 0 & 0 & 0\\ 0 & \alpha & \sqrt{\left(1/2-\alpha \right)\alpha } & 0\\ 0 & \sqrt{\left(1/2-\alpha \right)\alpha } & 1/2-\alpha & 0\\ 0 & 0 & 0 & 0 \end{array}\right]\;,$$

while the density matrix for a double excited system is equal to

(19) $$\rho_{ij}=\left[\begin{array}{cccc} 0 & 0 & 0 & 0\\ 0 & \alpha & \sqrt{\left(1/2-\alpha \right)\alpha } & 0\\ 0 & \sqrt{\left(1/2-\alpha \right)\alpha } & 1/2-\alpha & 0\\ 0 & 0 & 0 & 1/2 \end{array}\right]\;,$$

and α reaches values from zero to 1/2. When α=1/4, the linear entropy $E_{ij}=2/3$, and the degree of coherence $D_{ij}^{2}=1/4$. Whereas, if α is equal to 0 or 1/2, the linear entropy $E_{ij}=2/3$ and $D_{ij}^{2}=1/2$. For states defined by the density matrix (18) and (19), the linear entropy takes the following form:

(20) $$E_{ij}=\frac{8}{3}\left(\alpha -\alpha^{2}-{\left(1/2-\alpha \right)}^{2}+1/2-\alpha -2\alpha \left(1/2-\alpha \right)\right)=\frac{2}{3}\;,$$

and does not depend on $D_{ij}^{2}$. We note that this value is the maximal value of linear entropy obtained in analyzed families of states.

For the remaining values of degree of coherence $D_{ij}^{2}$ fulfilling relation $D_{ij}^{2}>0.5$, the density matrix $\rho_{ij}$ describing the states corresponding to the maximal values of the linear entropy for single excited states’ case is

(21) $$\rho_{ij}=\left[\begin{array}{cccc} 1-\alpha -\beta & 0 & 0 & 0\\ 0 & \alpha & 0 & 0\\ 0 & 0 & \beta & 0\\ 0 & 0 & 0 & 0 \end{array}\right]\;,$$

while, for the case of the double excitation, it takes the form

(22) $$\rho_{ij}=\left[\begin{array}{cccc} 0 & 0 & 0 & 0\\ 0 & \alpha & 0 & 0\\ 0 & 0 & \beta & 0\\ 0 & 0 & 0 & 1-\alpha -\beta \end{array}\right].$$

The full density matrix (describing three-qubit system) for such situations is

(23) $$\rho =\alpha |\psi_{1}\rangle \langle \psi_{1}\left|+\beta \right|\psi_{2}\rangle \langle \psi_{2}|+\left(1-\alpha -\beta \right)|\psi_{3}\rangle \langle \psi_{3}|,$$

where $\psi_{i}=\{|001\rangle ,|010\rangle ,|100\rangle \}$ and $\alpha ,\beta =\{P_{001},P_{010},P_{101}\}$ or $\psi_{i}=\{|011\rangle ,|101\rangle ,$|110〉} and $\alpha ,\beta =\{P_{011},P_{101},P_{110}\}$ for the system with single and double excitation, respectively, and one of the probabilities, α or β, equals zero. If α=0, the probability β can take values from zero to unity. When β is 0 or 1, the linear entropy reaches zero, and the degree of coherence is equal to 1—while, for β=1/2, the linear entropy $E_{ij}=2/3$ and $D_{ij}^{2}=1/2$.

In fact, the two-qubit states discussed here are the mixtures of two separable states. For such a case, the relation between the linear entropy and the degree of coherence derived for those density matrices using the Formulas (10)–(13) can be expressed as

(24) $$E_{ij}=\frac{4}{3}-\frac{4}{3}D_{ij}^{2}\;,$$

which is represented by the dash-dotted line in Figure 2.

In the following steps, we will derive the formula determining the boundary values of linear entropy parametrized by the degree of coherence for the strongly entangled states. In Figure 2b, the red area corresponds to such states, and the dotted line presents such boundary values of linear entropy.

To determine the degree of entanglement between two qubits, we will apply the *concurrence*. The concurrence of the qubit–qubit subsystem can be calculated with the application of the definition proposed by Hill and Wootters [75,76]

(25) $$C_{ij}=C\left(\rho_{ij}\right)=max\left(\sqrt{\lambda_{I}}-\sqrt{\lambda_{II}}-\sqrt{\lambda_{III}}-\sqrt{\lambda_{IV}},0\right)\;,$$

where the parameters $\lambda_{l}$ are the eigenvalues of matrix *R* obtained from the relation $R=\rho_{ij}\tilde{\rho_{ij}}$, $\tilde{\rho_{ij}}$ is defined as $\tilde{\rho_{ij}}=\sigma_{y}⊗\sigma_{y}\rho_{ij}^{*}\sigma_{y}⊗\sigma_{y}$, and $\sigma_{y}$ is a 2×2 Pauli matrix.

Next, applying definition (25), we derive the formulas describing concurrence for different pairs of qubits. For the systems with single excitation, concurrence can be expressed by the probabilities as:

(26) $$\begin{array}{ccc} C_{12} & = & \sqrt{4P_{100}P_{010}}\;,\\ C_{13} & = & \sqrt{4P_{100}P_{001}}\;,\\ C_{23} & = & \sqrt{4P_{010}P_{001}}\;, \end{array}$$

and, for the double excited system, is

(27) $$\begin{array}{ccc} C_{12} & = & \sqrt{4P_{011}P_{101}}\;,\\ C_{13} & = & \sqrt{4P_{011}P_{110}}\;,\\ C_{23} & = & \sqrt{4P_{101}P_{110}}\;. \end{array}$$

In the next step, we shall identify states that are strongly entangled. In our consideration, we assume that the strongly entangled states are those for which the concurrence takes values equal to or higher than 0.9. Applying definition (27,28) and assuming that $C_{ij}=0.9$, we can find the relations among probabilities $P_{ijk}$ and obtain the formula that gives the value of the linear entropy represented in Figure 2b by the dotted line:

(28) $$E_{ij}=\frac{19}{75}-\frac{4}{3}D_{ij}^{2}.$$

From Figure 2b, we see that the two-qubit states are strongly entangled when the linear entropy and degree of coherence reach small values. More precisely, the strongly entangled states (when $C_{ij}\geq 0.9$) can be generated when the linear entropy becomes equal to or smaller than those defined by Equation (28) for $D_{ij}^{2}\in \langle 0.01;0.19\rangle$ and when $D_{ij}^{2}<0.01$ by Formula (17).

In three-qubit systems, in addition to entanglement between two qubits, we can also analyze the entanglement of one qubit with the other two. Such entanglement can be quantified by the bipartite concurrence [77]

(29) $$C_{k-ij}=\sqrt{2-2Tr\left(\rho_{k}^{2}\right)}\;,$$

where $\rho_{k}$ is the reduced density matrix related to qubit *k*, and the quantity $C_{k-ij}$ describes entanglement between qubit *k* and pair of qubits *i* and *j*.

The families of states analyzed here are W-class states. For such states, the three-tangle $\tau_{ijk}$ that describes the three-way entanglement vanishes. Therefore, using the definition of three-tangle [77],

(30) $$\tau_{ijk}=C_{k-ij}^{2}-C_{ik}^{2}+C_{jk}^{2}\;,$$

we can write the monogamy relation in the following form:

(31) $$C_{k-ij}^{2}=C_{ik}^{2}+C_{jk}^{2}.$$

The relation (31) can be confirmed using Equations (27), (28) and (29), and is in agreement with the results presented in [77].

Next, applying formulas (10), (12), (27), (28) and (31), we can find the relation between linear entropy $E_{ij}$ and concurrence $C_{k-ij}$:

(32) $$E_{ij}=\frac{2}{3}C_{k-ij}^{2}.$$

Analyzing Equations (27) and (28), we find that maximal value of $C_{ik}^{2}$ parametrized by $C_{jk}^{2}$ is

(33) $$\max C_{ik}^{2}=1-C_{jk}^{2}\;,$$

and the maximal reachable value by concurrence $C_{k-ij}$ is 1. Therefore, based on Equation (32), we can confirm that the maximal value of linear entropy obtained in analyzed families of states is 2/3.

## 4. The First-Order Correlation Function and Linear Entropy

In Section 3, we discussed the relationship between the internal coherence of subsystems (quantified by the degree of coherence $D_{ij}^{2}$), linear entropy and concurrence. Here, we shall consider the relationships among the mutual coherence quantified by the first-order correlation function and linear entropy and concurrence. Such first-order cross-correlation function for subsystems *i* and *j* can be written as [78,79]:

(34) $$g_{ij}^{\left(1\right)}=\frac{\left|\langle {\hat{a}}_{i}^{†}{\hat{a}}_{j}\rangle \right|}{\sqrt{\langle {\hat{a}}_{i}^{†}{\hat{a}}_{i}\rangle \langle {\hat{a}}_{j}^{†}{\hat{a}}_{j}\rangle }}.$$

The function $g_{ij}^{\left(1\right)}$ can take values from zero to unity. For maximally coherent states, it equals 1, whereas, when we do not observe coherence between subsystems *i* and *j*, $g_{ij}^{\left(1\right)}=0$.

All states corresponding to the single excitation’s case, described by the wave function (1), are fully coherent and thus $g_{ij}^{\left(1\right)}=1$. In contrast, if we assume the presence of two excitations (see, the wave function (3), the first-order correlation function can take various values from 0 to 1. Therefore, in further analysis, we focus only on the relations between linear entropy and first-order coherence for double excited systems.

**Figure 3:**
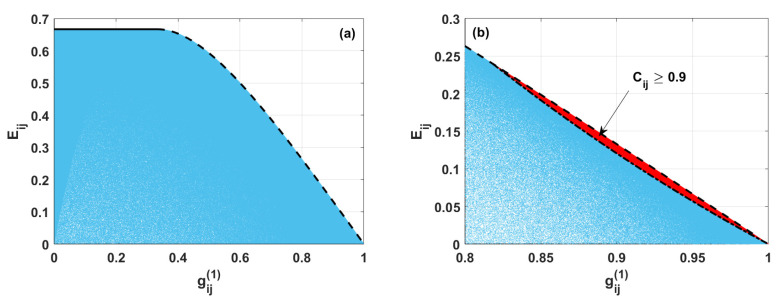
(**a**) Linear entropy $E_{ij}$ versus first-order correlation function $g_{ij}^{\left(1\right)}$ for two-qubit states described by the density matrix $\rho_{ij}$, calculated numerically (blue area). Black lines are plotted according to the analytical formulas derived here determining the borders between various regions of the states. (**b**) The same as in (**a**). Additionally, the red area presents the possible values of linear entropy and the first-order correlation function for two-qubit states with concurrence $C_{ij}>0.9$ (red area).

In Figure 3, we present the results of numerical analysis concerning the ensemble of randomly generated states describing double excited systems. For such states, the blue area shows possible values of linear entropy for given values of the first-order correlation function. The boundary values of linear entropy are represented by black lines: solid and dashed ones.

To derive the maximal values of linear entropy parametrized by the first-order correlation function, we find the formulas describing $g_{ij}^{\left(1\right)}$ function expressed by probabilities:

(35) $$\begin{array}{ccc} g_{12}^{\left(1\right)} & = & \frac{C_{011}^{*}C_{101}}{\sqrt{\left(P_{101}+P_{110}\right)\left(P_{011}+P_{110}\right)}}\;,\\ g_{13}^{\left(1\right)} & = & \frac{C_{011}^{*}C_{110}}{\sqrt{\left(P_{110}+P_{101}\right)\left(P_{011}+P_{101}\right)}}\;,\\ g_{23}^{\left(1\right)} & = & \frac{C_{101}^{*}C_{110}}{\sqrt{\left(P_{110}+P_{011}\right)\left(P_{101}+P_{011}\right)}}\;. \end{array}$$

In further analysis, we will consider real probability amplitudes $C_{ijk}^{*}=C_{ijk}=\sqrt{P_{ijk}}$.

From Figure 3, we see that, for $g_{ij}^{\left(1\right)}\leq 1/3$, the maximal value of $E_{ij}$ does not depend on the value of the first-order correlation function. For such a case, the two-qubit matrix is expressed by Equation (19), and the corresponding first-order correlation function is given as

(36) $$g_{ij}^{\left(1\right)}=\frac{\sqrt{\left(1/2-\alpha \right)\alpha }}{\sqrt{\left(1-\alpha \right)\left(\alpha +1/2\right)}}.$$

Thus, for $g_{ij}^{\left(1\right)}\leq 1/3$, the maximal value of $E_{ij}$ is equal to 2/3 and does not depend on the values of the parameter α.

From the other side, when $g_{ij}^{\left(1\right)}>1/3$, the maximal possible value of linear entropy decreases with the increasing value of the first-order correlation function (see the dashed line in Figure 3). In such a case, the density matrix describing the system is:

(37) $$\begin{array}{ccc} \rho_{ij} & = & \left[\begin{array}{cccc} 0 & 0 & 0 & 0\\ 0 & \alpha & \sqrt{\alpha \beta } & 0\\ 0 & \sqrt{\alpha \beta } & \beta & 0\\ 0 & 0 & 0 & 1-\alpha -\beta \end{array}\right], \end{array}$$

where the probabilities α and β have to be equal to

(38) $$\alpha =\beta =\frac{g_{ij}^{\left(1\right)}}{1+g_{ij}^{\left(1\right)}}\;,$$

and the probabilities α and β can take values within the range 〈1/4,1/2〉. When α=β=1/4, the first-order correlation function is 1/3, and $E_{ij}=2/3$. However, if α=β=1/2, the linear entropy reaches zero, and function $g_{ij}^{\left(1\right)}$ is equal to unity.

In general, for the two-qubit states represented by Equation (37), $E_{ij}$ fulfills the following relation:

(39) $$E_{ij}=-\frac{16\left(g_{ij}^{\left(1\right)}-1\right)g_{ij}^{\left(1\right)}}{3{\left(1+g_{ij}^{\left(1\right)}\right)}^{2}}.$$

In the next step, we discuss the case when the states are strongly entangled ones, i.e., the concurrence is assumed to be equal to or higher than 0.9. For such a situation, the minimal value of $E_{ij}$ parametrized by $g_{ij}^{\left(1\right)}$ is defined by the condition represented by the dash-dotted line in Figure 3b. The red area corresponds to the values linear entropy and first-order correlation function for states presenting strong entanglement. From Figure 3b, we see that the states with $C_{ij}\geq 0.9$ exhibit a high level of the first-order correlation function $g_{ij}^{\left(1\right)}\in \langle 9/11;1\rangle$. Moreover, for such the case, the linear entropy is limited to values determined by:

(40) $$E_{ij}\geq -\frac{27\left(g_{ij}^{{\left(1\right)}^{2}}-1\right)\left(481g_{ij}^{{\left(1\right)}^{2}}-81\right)}{20000g_{ij}^{{\left(1\right)}^{4}}}.$$

We derived that condition using the definitions (12), (28) and (36) and assuming that $C_{ij}=0.9$.

Thus, one can state that the strongly entangled two-qubit states are simultaneously characterized by low levels of mixedness and high values of the first-order coherence function.

## 5. The Second-Order Correlation Function and Linear Entropy

Analogously, as in the previous section, we will analyze at this point relations between the degree of mixedness and second-order coherence function $g_{ij}^{\left(2\right)}$. This function quantifies the correlations between intensities of field, contrary to $g_{ij}^{\left(1\right)}$ considered earlier that described the correlations between the amplitudes of two fields. $g_{ij}^{\left(2\right)}$ is defined here for two subsystems *i* and *j* and can be expressed as [78,79]:

(41) $$g_{ij}^{\left(2\right)}=\frac{\langle {\hat{a}}_{i}^{†}{\hat{a}}_{j}^{†}{\hat{a}}_{i}{\hat{a}}_{j}\rangle }{\langle {\hat{a}}_{i}^{†}{\hat{a}}_{i}\rangle \langle {\hat{a}}_{j}^{†}{\hat{a}}_{j}\rangle }.$$

Applying the procedure described in the previous section, we shall concentrate here on the case of double excited systems described by the density matrix (4). For such a situation, the second-order correlation function expressed by probabilities for each qubit–qubit subsystem can be written as:

(42) $$\begin{array}{ccc} g_{12}^{\left(2\right)} & = & \frac{P_{110}}{\left(P_{101}+P_{110}\right)\left(P_{011}+P_{110}\right)}\;,\\ g_{13}^{\left(2\right)} & = & \frac{P_{011}}{\left(P_{110}+P_{101}\right)\left(P_{011}+P_{101}\right)}\;,\\ g_{23}^{\left(2\right)} & = & \frac{P_{101}}{\left(P_{110}+P_{011}\right)\left(P_{101}+P_{011}\right)}\;. \end{array}$$

**Figure 4:**
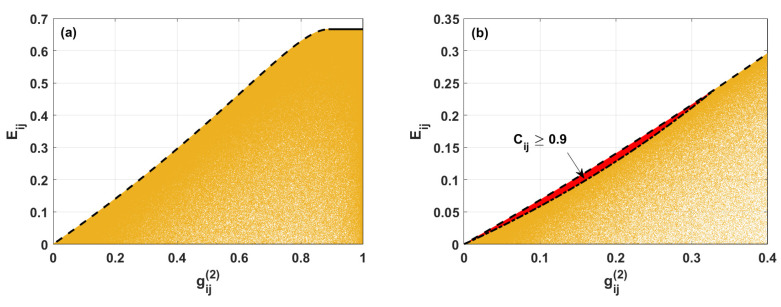
(**a**) Linear entropy $E_{ij}$ versus second-order correlation function $g_{ij}^{\left(2\right)}$ for two-qubit states described by the density matrix $\rho_{ij}$, calculated numerically (yellow area). Black lines are plotted according to the analytical formulas derived here and determining the borders between various regions of the states. (**b**) The same as in (**a**). Additionally, the red area presents the possible values of linear entropy and the second-order correlation function for two-qubit states with concurrence $C_{ij}>0.9$ (red area).

Figure 4 depicts numerical results of analysis of randomly generated states for the system with double excitation. The same as previously, colored areas correspond to the possible achievable states characterized by various pairs of the values of the linear entropy and $g_{ij}^{\left(2\right)}$. The black lines appearing there denote the boundary values of the entropy for the particular $g_{ij}^{\left(2\right)}$. When $g_{ij}^{\left(2\right)}<8/9$, the maximal possible value of $E_{ij}$ monotonously increases with the increasing value of the second-order correlation function (see the dashed line in Figure 4). In such a case, using Equations (12) and (43), we find that the maximal value of $E_{ij}$ fulfills the relation:

(43) $$E_{ij}=\frac{16{\left(\sqrt{1-g_{ij}^{\left(2\right)}}-1\right)}^{2}\left(\sqrt{1-g_{ij}^{\left(2\right)}}+g_{ij}^{\left(2\right)}-1\right)}{3g_{ij}^{{\left(2\right)}^{2}}}.$$

The entropy $E_{ij}$ given by (43) reaches its maximal values when the system is described by the density matrix (37) with the probabilities α and β equal to:

(44) $$\alpha =\beta =\frac{g_{ij}^{\left(2\right)}+\sqrt{1-g_{ij}^{\left(2\right)}}-1}{g_{ij}^{\left(2\right)}}$$

where α and β can take values in the range 〈0;1/2〉. When both α and β are simultaneously equal to 0 or 1/2, the second-order correlation function and entropy become equal to zero. However, if α=β=1/4, the linear entropy $E_{ij}=2/3$, and $g_{ij}^{\left(2\right)}$ reaches =8/9.

However, when $g_{ij}^{\left(2\right)}\geq 8/9$, the maximal possible value of linear entropy stops being dependent on the second-order correlation function and remains equal to 2/3 (see the black solid line in Figure 4. For such a case, the two-qubit density matrix is described by Equation (19).

In Figure 4b, the red area corresponds to the strongly entangled states with concurrence $C_{ij}\geq 0.9$. The dash-dotted line appearing there represents the condition for the minimal values of $E_{ij}$ parametrized by $g_{ij}^{\left(2\right)}$. Simultaneous analysis of Equations (12), (28) and (43), describing the entropy, second-order correlation function, concurrence, respectively, and assuming that concurrence is equal to 0.9 gives us the minimal achievable entropy for strongly entangled states:

(45) $$E_{ij}=\frac{27\left(400-481g_{ij}^{\left(2\right)}\right)g_{ij}^{\left(2\right)}}{20000{\left(g_{ij}^{\left(2\right)}-1\right)}^{2}}\;,$$

where $g_{ij}^{\left(2\right)}\in \langle 0;40/121\rangle$. It is seen that the strongly entangled two-qubit states are characterized by simultaneously low levels of both mixedness and second-order coherence function.

## 6. Conclusions

In this work, we have analyzed two families of three-qubit states in the context of the appearance of coherence and entanglement as quantum resources, and the mixedness of discussed states. In particular, we have focused on the characteristics of possible achievable states describing the two-qubit subspace of the system. Applying the tracing out procedure, we have analyzed the degree of mixedness of such two-qubit states, the bipartite coherences, and entanglement. We have compared the degree of mixedness and the parameters describing coherences, such as the degree of coherence, the first- and second-order correlation function, and have shown the relations among them. Based on such performed analysis, we have derived boundary conditions for possible achievable strongly entangled two-qubit states. We have shown that the strongly entangled states can be characterized by low levels of mixedness and degree of coherence. On the other hand, analyzing the correlation functions $g_{ij}^{\left(1\right)}$ and $g_{ij}^{\left(2\right)}$, it turned out that highly entangled states are states with high and low levels of the first and second-order correlation function, respectively.

## Data Availability

Not applicable.
